# Liver fatty acid binding protein expression in colorectal neoplasia

**DOI:** 10.1038/sj.bjc.6601828

**Published:** 2004-04-27

**Authors:** L C Lawrie, S R Dundas, S Curran, G I Murray

**Affiliations:** 1 Department of Pathology, University of Aberdeen, Foresterhill, Aberdeen, Foresterhill AB25 2ZD, UK

**Keywords:** colon adenoma, colon cancer, 2D gel electrophoresis, immunohistochemistry

## Abstract

Liver fatty acid binding protein is a member of the fatty acid binding group of proteins that are involved in the intracellular transport of bioactive fatty acids and participate in intracellular signalling pathways, cell growth and differentiation. In this study we have used proteomics and immunohistochemistry to determine the changes in liver fatty acid binding protein in colorectal neoplasia. Comparative proteome analysis of paired samples colorectal cancer and normal colon identified consistent loss of liver fatty acid binding protein (L-FABP) in colorectal cancer compared with normal colon. To identify the changes in liver fatty acid binding protein expression during colorectal cancer development and progression the cell-specific expression of L-FABP was determined by immunohistochemistry in a series of colorectal cancers and colorectal adenomas. Decreased L-FABP immunoreactivity was significantly associated with poorly differentiated cancers (*P*<0.001). In colorectal adenomas there was a significant trend towards decreased staining of L-FABP in the larger adenomas (*P*<0.001). There was consistent L-FABP immunostaining of normal surface colonocytes. This study demonstrates that loss of L-FABP occurs at the adenoma stage of colorectal tumour development and also indicates that L-FABP is a marker of colorectal cancer differentiation.

The fatty acid binding proteins are a group of low molecular weight proteins involved in the intracellular transport of long-chain bioactive fatty acids including linoleic acid and its derivatives such as arachidonic acid (Glatz and van der Vusse, 1996; Hertzel and Bernlohr, 1998). The designation of each of these proteins has been derived from the tissue from which it was originally isolated and key members of this group of proteins include liver fatty acid binding protein, intestinal fatty acid binding protein and epidermal fatty acid binding protein. Individual fatty acid binding proteins show specific lipid-binding profiles and distinct patterns of tissue and cell-type specific expression (Veerkamp *et al*, 1999; Richieri *et al*, 2000; Duplus and Forest, 2002). Specific regulatory mechanisms operate for each fatty acid binding protein.

These proteins play a central role in the solubilisation and the intracellular compartmentalisation of fatty acids, thus facilitating the cellular uptake and intracellular trafficking and processing of fatty acids (Veerkamp *et al*, 1999; Storch and Thumser, 2000). Fatty acid binding proteins play an active part in fatty acid-mediated signal transduction pathways and regulation of gene expression (Hertzel and Bernlohr, 1998). As a result, fatty acid binding proteins are involved in modulating cell division (Sorof, 1994), cell growth and differentiation (Schroeder *et al*, 2001) and also by preventing high intracellular fatty acid concentrations, protect cells against the cytotoxic effects of fatty acids (Glatz *et al*, 1997).

In view of the role of fatty acid binding proteins in a variety of key physiological functions it has been proposed that the alterations that occur in individual fatty acid binding protein expression during tumour development and progression (Celis *et al*, 1996; Jing *et al*, 2000) may contribute to tumorigenesis. Additionally, it has been suggested that the expression of individual fatty acid binding proteins in tumours may also serve as useful diagnostic markers and novel therapeutic targets (Das *et al*, 2001).

We have been performing proteomic studies to identify changes in protein expression in colorectal cancers (Lawrie and Murray, 2002) and have discovered that the expression of one member of the family of fatty acid binding proteins namely liver fatty acid binding protein (L-FABP) is consistently lost in colorectal cancer. The cell-specific expression of L-FABP has been determined using immunohistochemistry in normal colon, adenomas and carcinomas. Examining the expression of L-FABP at different stages of tumour development reveals that L-FABP shows loss at the adenoma stage of colorectal tumour development.

## MATERIALS AND METHODS

### Tissue samples

Proteomic analysis was performed on 20 matched pairs of Dukes' C colorectal cancers and normal colorectal tissue samples obtained from primary colorectal cancer resections and which had been stored in the Aberdeen colorectal tumour bank. None of the patients in this study had received any chemotherapy or radiotherapy prior to surgery. The project had the approval of the local research ethics committee. Representative samples of viable tumour and normal colorectal mucosa (obtained at least 5 cm from tumour) were dissected from colorectal cancer excision specimens within 30 min of surgical removal. Dissected samples were immediately frozen in liquid nitrogen and stored at −80°C prior to analysis (Table 1

**Table 1:** Clinicopathological characteristics of the cases used for proteome analysis. All the cases were Dukes' C colorectal cancers

**Characteristic**	**Number**
Gender	Male, *n*=10
	Female, *n*=10
	
Age	<55 years, *n*=7
	⩾55 years, *n*=13
	
Site	Proximal, *n*=9
	Distal, *n*=10
	Rectum, *n*=1
	
Tumour differentiation	Well, *n*=0
	Moderate, *n*=19
	Poor, *n*=1

).

Immunohistochemistry for L-FABP was performed on formalin fixed wax embedded colorectal cancer samples obtained from patients who had undergone elective surgery for colorectal cancer (*n*=249) and were also selected from the Aberdeen colorectal tumour bank. These cases have been extensively phenotyped for a range of markers as previously described (McKay *et al*, 2000, 2002; Leeman *et al*, 2002) and include the samples used for proteome analysis. Detailed clinico-pathological data (Dukes’ stage, degree of primary tumour differentiation, site of primary tumour, patient age, and gender) are shown in Table 2

**Table 2:** Clinico-pathological characteristics of the colorectal cancer cases studied by immunohistochemistry

**Characteristic**	**Number (%)**
*Gender*	
Male	133 (53.4)
Female	116 (46.6)
	
*Age, years*	
Range	32–91
Mean	68
Median	69
	
*Site*^a^	
Proximal colon	90 (36.1%)
Distal colon	99 (39.8%)
Rectum	60 (24.1%)
	
*Dukes' stage*	
A	32 (12.9%)
B	123 (49.4%)
C	94 (37.7%)
	
*Tumour differentiation*	
Well	8 (3.2%)
Moderate	205 (82.3%)
Poor	36 (14.5%)

a Proximal colon tumours arose proximal to the splenic flexure, and distal colon tumours arose distal to this site.

. Immunohistochemistry was also performed on formalin fixed wax embedded sections of colorectal adenomas (*n*=38 consisting of 19 tubular adenomas, 16 tubulo-villous adenomas and three villous adenomas; six adenomas less than 5 mm in size, 16 adenomas of 5–10 mm in size and 16 adenomas greater than 10 mm in size).

## TWO-DIMENSIONAL GEL ELECTROPHORESIS

### Lysis buffer preparation

Lysis buffer was prepared according to our established protocols (Lawrie *et al*, 2001a) and contained urea (42% w v^−1^); thiourea (15% w v^−1^); Chaps [3-(3-cholamidopropyl)dimethylammonio-1-propanesulphonate] (4% w v^−1^); *N*-decanoyl-*N*-methylglucamine (Mega 10, 1% w v^−1^), 1-*O*-Octyl-*β*-D-glucopyranoside (OBG, 1% w v^−1^), Triton X-100 (polyoxyethylene-*p*-isooctylphenol) (0.5% v v^−1^); Tris [Tris(hydroxymethyl)aminomethane] (0.5% w v^−1^); DTT (dithiothreitol) (0.8% w v^−1^); IPG 3-10 NL (immobilised pH gradient) buffer (1% v v^−1^), *β*-mercaptoethanol (1% v v^−1^), tributylphosphine (0.02% v v^−1^). All chemicals were obtained from Amersham Biosciences, UK, with the exception of OBG (Aldrich, UK) and Mega 10 (Sigma, UK).

### Isoelectric focusing

Frozen sections (10 *μ*m in thickness) of tumour and normal were cut using a cryostat and 30 10-*μ*m sections of normal tissue or 30 10-*μ*m sections of tumour tissue were solubilised in lysis buffer (Lawrie *et al*, 2001a). One section from each tumour and normal sample was stained with haematoxylin and eosin to confirm the morphology; an assessment of tumour cellularity was also made of each tumour sample to ensure that each of those samples contained a high proportion of tumour cells. In total, 500 *μ*g of normal and tumour sample, respectively, were loaded, in duplicate, into Immobiline Dry strip holders and Immobiline Drystrips, pI 3-10 NL (Amersham Biosciences), were placed into the strip holders. Strips were incubated overnight at room temperature to allow sample absorption. After incubation strips were removed, and strip holders were cleaned and small pieces of dampened electrode strips were then placed over the electrodes in the strip holders to help absorb excess salt during the first dimension focusing stage. The strips were then placed back into the strip holders and covered with dry strip cover fluid (Amersham Biosciences).

The first dimension focusing was carried out on an IPGPhor system under the following conditions: 30 min at 20 V, 1.5 h at 200 V, 1.5 h gradient to 3500 V, 35 h at 3500 V, at 15°C. After completion of focusing the strips were equilibrated for 30 min in equilibration buffer containing urea (36% w v^−1^); 0.5 M Tris-HCl, pH 6.9 (20% v v^−1^); 20% SDS (dodecyl sulphate, sodium salt) (20% v v^−1^); DTT (0.4% w v^−1^); glycerol (30% v v^−1^). Strips were equilibrated for a further 30 min in equilibration buffer where DTT was replaced by iodoacetamide (1% w v^−1^). All chemicals were obtained from Amersham Biosciences.

Proteins were separated in the second dimension according to their molecular weight using 7 cm NuPAGE 4–12%, 1 well, Bis-Tris gel (Invitrogen, Paisley, UK). First dimension strips were attached to the second dimension gel with a 4% low melting point agarose solution (Amersham Biosciences). Normal and tumour samples from the same patient were run in the same gel tank to account for any differences caused by gel electrophoresis. Gels were run at a constant 120 V until the bromophenol dye front reached the end of the gel.

Proteins were visualised using a Colloidal Blue Staining Kit (Invitrogen, Paisley, UK) used according to the manufacturer's protocol.

### Detection of differential protein expression

Gels were destained using HPLC-grade water with microwave heating. Destained gels were immediately photographed to produce a black and white image. Gel photographs were scanned to produce a computer image, which was then enlarged and printed onto sheets of acetate. Overlaying the normal and tumour acetate gel pictures allowed proteins that were differentially expressed to be detected. Differentially expressed protein spots were cut from the gel in preparation for identification by mass spectrometry.

### Identification of differentially expressed proteins

Individual proteins were identified by peptide mass mapping. Protein spots were cut from the gel, washed to remove Coomassie stain, reduced with DTT, alkylated with iodoactetamide and then digested with trypsin. The resultant tryptic peptides were extracted from the gel pieces under full automation (Pro-Gest Robot, Genomic Solutions), desalted using microporous tips (Millipore) deposited onto a sample plate along with a matrix chemical (*α*-cyano-4-hydroxycinnamic acid) under full automation (Pro-MS, Genomic Solutions). The masses of the tryptic fragments were determined by Matrix Assisted Laser Desorption Ionisation Time of Flight Mass Spectrometry (MALDI-TOF MS) using a PerSeptive Biosystems Voyager-DE STR mass spectrometer. To identify the original protein, the masses of the tryptic peptides were entered into the MS-Fit database-searching program. The database search was restricted to human proteins with no constraints on either the molecular weight or the isoelectric point of the protein. A clear difference in statistical score between the proteins ranked first and second in the results list had to be obtained in order to confirm that the correct protein was identified.

### Immunohistochemistry

Immunohistochemistry was performed using a mouse monoclonal antibody to L-FABP (Novocastra, Newcastle-upon-Tyne, UK) at a dilution of 1 : 200. A Dako TechMate™ 500 autostainer was used to immunostain 4 *μ*m thick formalin fixed wax embedded sections with the L-FABP antibody. A prior antigen retrieval step in boiling 10 mM citrate buffer pH 6 for 20 min was performed. Biotinylated goat anti-mouse secondary antibody, a complex of avidin with horseradish peroxidase (HRP) and diaminobenzidine (DAB) were used as the detection-staining system. The cellular localisation of L-FABP immunoreactivity was determined and scored for both intensity (negative, weak, moderate and strong) and proportion (0, 1–5, 6–25, 26–50, 51–75 and 76–100%) of stained cells as previously described (Leeman *et al*, 2002; McKay *et al*, 2002). To allow comparison of L-FABP immunostaining with pathological stage and survival for each cancer integer values were assigned to the scores of intensity (0–3) and proportion of tumour cells stained (0–5). These values were multiplied together to provide a single L-FABP score as previously described (Leeman *et al*, 2002). Statistical analysis was performed with SPSS v10 for Windows 95™.

## RESULTS

### Proteomic analysis

**Figure 1:**
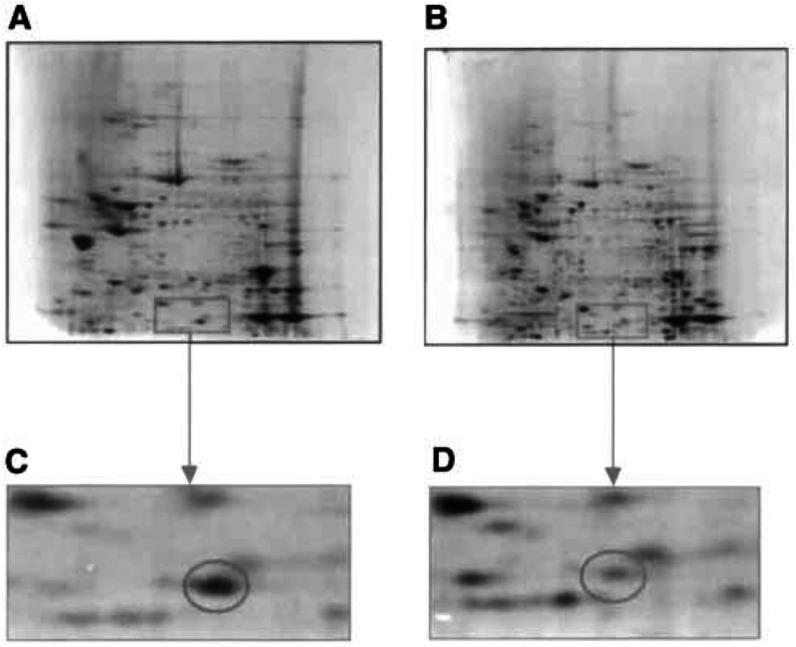
Representative 2D gel showing the proteins expressed by a sample of (**A**) normal colon tissue, (**B**) colorectal cancer tissue. A section of each of the normal (**C**) and tumour (**D**) gels have been enlarged to show the loss of L-FABP in the tumour sample.

Protein expression profiles were generated from 20 pairs, in duplicate, of normal mucosa and Dukes’ C colorectal cancers. Protein profiles were reproducible with replicate samples taken from the individual patients, but also when profiles were compared with samples taken from other patients. Between 400 and 500 distinct proteins spots could be detected on each colloidal Coomassie blue stained mini gel (Figure 1

).

L-FABP was found to be consistently decreased in all 20 tumour samples. The masses of the tryptic peptides were entered into the MS-Fit database searching program, the results of which can be seen in Table 3

**Table 3:** Identification of L-FABP

**MOWSE score**	**Number of matching peptides**	**Percent coverage**	**Protein MW (Da)**	**Protein pI**	**SwissProt Accession no.**	**Protein name**
1045000	18	96	14 209	6.6	P07148	Liver fatty acid- binding protein
462	4	32	18 013	7.7	P05092	Peptidyl-prolyl *cis*–*trans* isomerase A
361	4	43	12 902	6.2	P23763	Vesicle-associated membrane protein 1

Example of an MS-Fit database search showing the identification of L-FAPB. The MOWSE score is much higher (225 times higher) for L-FABP compared with the next protein score. The ‘MOWSE score’ is a score based on how many peptides have been matched to the protein in the database and the accuracy of those matches, the higher the MOWSE score the better the hit; ‘no. of matching peptides’ is the number of experimentally obtained peptides that have been matched to theoretical peptides in the database; ‘Percent coverage’ is the percentage of the protein sequence covered by the matched peptides. ‘Protein MW and pI’ are the theoretical molecular weights and isoelectric points of the proteins; ‘Swissprot Accession no’ is an accession number in the SwissProt database that allows further information to be obtained about the identified protein; ‘protein name’ shows the name of the identified protein.

. In the example data shown, 18 of the experimentally obtained peptides were matched, with better than 50 ppm accuracy, to the sequence of L-FABP, which resulted in a coverage of 96% of the L-FABP amino-acid sequence. From the ‘in-depth’ database search results (Table 4

**Table 4:** Detailed mass spectrometric analysis of L-FABP

**m/z submitted**	**MH^+^ matched**	**Delta (ppm)**	**Modifications**	**Start**	**End**	**Missed cleavages**	**Database sequence**
1210.70	1210.70	0		21	31	0	AIGLPEELIQK
1442.77	1442.78	5		37	49	1	GVSEIVQNGKHFK
1752.00	1752.03	17		21	36	2	AIGLPEELIQKGKDIK
1762.79	1762.81	12		7	20	0	YQLQSQENFEAFMK
1778.80	1778.81	1	1Met-ox	7	20	0	YQLQSQENFEAFMK
1791.92	1791.99	38		81	96	1	TVVQLEGDNKLVTTFK
2019.12	2019.15	16		79	96	2	VKTVVQLEGDNKLVTTFK
2147.11	2147.21	47		81	99	2	TVVQLEGDNKLVTTFKNIK
2248.15	2248.21	28		37	57	2	GVSEIVQNGKHFKFTITAGSK
2311.11	2311.07	18	AcetN	1	20	1	SFSGKYQLQSQENFEAFMK
2327.12	2327.07	25	AcetN 1Met-ox	1	20	1	SFSGKYQLQSQENFEAFMK
2381.13	2381.23	41		100	121	0	SVTELNGDIITNTMTLGDIVFK
2443.06	2443.10	18		58	78	0	VIQNEFTVGEECELETMTGEK
2537.33	2537.33	0		100	122	1	SVTELNGDIITNTMTLGDIVFKR
2553.28	2553.32	16	1Met-ox	100	122	1	SVTELNGDIITNTMTLGDIVFKR
2670.23	2670.26	13		58	80	1	VIQNEFTVGEECELETMTGEKVK
2686.37	2686.26	40	1Met-ox	58	80	1	VIQNEFTVGEECELETMTGEKVK
2970.50	2970.49	4	1Met-ox	7	31	1	YQLQSQENFEAFMKAIGLPEELIQK

An example of the MS-Fit database ‘in depth’ search results where ‘m/z submitted’ is the experimentally obtained tryptic masses, ‘MH^+^ matched’ is the theoretical tryptic mass obtained after theoretical digestion of L-FABP, which the experimental tryptic mass has been matched to, ‘delta ppm’ is the difference between the experimentally and theorectically obtained tryptic masses. ‘Modifications’ identifies the modification on any peptide, where 1Met-ox is the oxidation of one methionine residue, and AcetN is the acetylation of the N-terminus. ‘start’ & ‘end’ identifies the position of the tryptic peptide within the protein sequence. ‘Missed cleavages’ identifies how many times trypsin missed a cleavage site. ‘Database sequence’ shows the sequence of the tryptic fragment matched in the database.

) it was also observed that the N-terminus of L-FABP was acetylated.

## L-FABP IMMUNOHISTOCHEMISTRY

### Colorectal carcinomas

In colorectal cancers L-FABP was observed in tumour cells while no immunoreactivity was identified in stromal cells, endothelial cells or inflammatory cells. The distribution of intensity and proportion of cells showing L-FABP immunoreactivity is summarised in Tables 5

**Table 5:** The proportion of tumour cells in colorectal cancers showing L-FABP immunoreactivity

**Proportion of L-FABP positive tumour cells**	**Percentage (number) of tumours**
Negative	18.5% (*n*=46)
1–5%	13.3% (*n*=33)
6–25%	20.1% (*n*=50)
26–50%	19.3% (*n*=48)
51–75%	23.3% (*n*=58)
76–100%	5.6% (*n*=14)

and 6

**Table 6:** Intensity of L-FABP immunostaining in colorectal cancer

**Intensity**	**Percentage (number) of tumours**
Negative	18.5% (*n*=46)
Weak	8.4% (*n*=21)
Moderate	20.5% (*n*=51)
Strong	52.6% (*n*=131)

**Figure 2:**
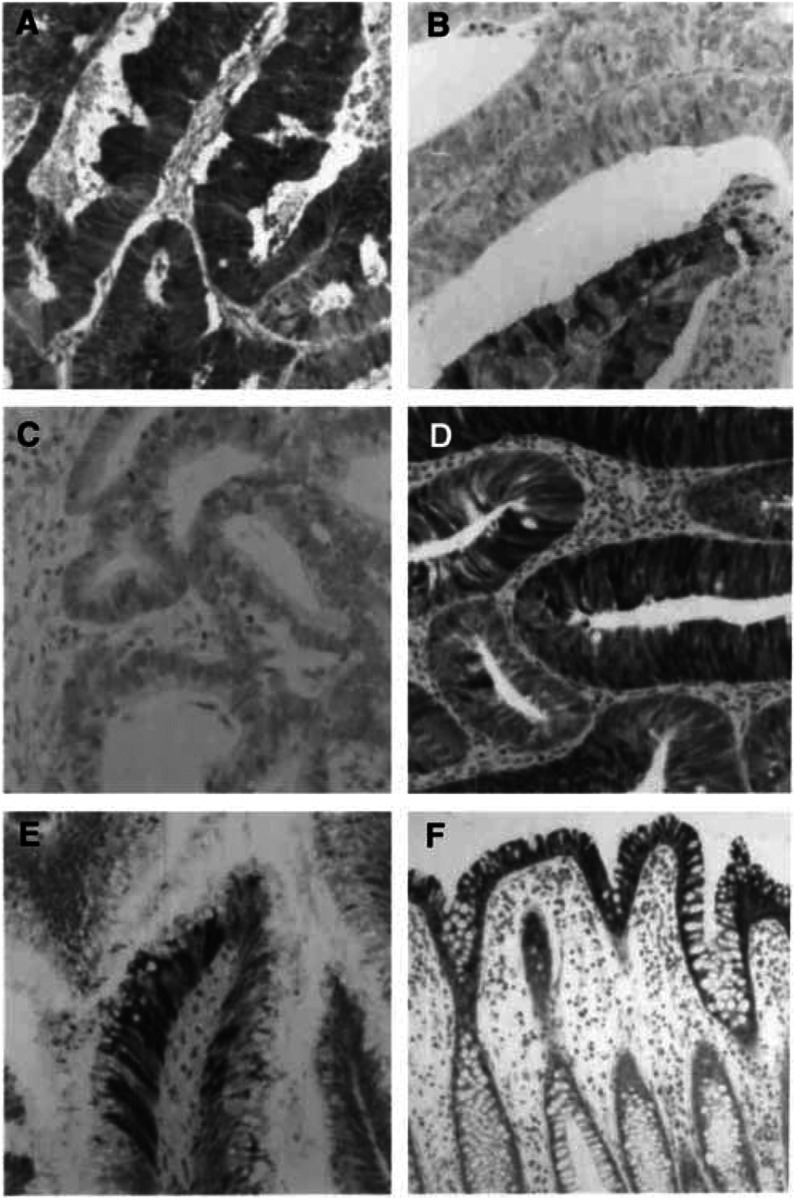
The immunohistochemical localisation of L-FABP in colon cancer, colon adenomas and normal colon. In colon cancer there is patchy staining of the tumour cells. (**A**) Tumour with a high proportion of tumour cells staining positively for L-FABP while (**B**) is a tumour with a low proportion of L-FABP positive tumour cells. The patchy or mosaic staining for L-FABP is also demonstrated in (**B)**. (**C**) Tumour that is negative for L-FABP. A small tubular adenoma (**D**) showing patchy staining for L-FABP. In a villous adenoma (**E**) there is only a small proportion of tumour cells showing L-FABP immunoreactivity and the staining is in discrete groups of cells. Immunohistochemical staining for L-FABP in normal colon is present in the surface epithelium and the upper half of the crypt epithelium (**F**).

. In those tumours that showed positive tumour cell staining the immunoreactivity for L-FABP usually occurred in patches or clusters (Figure 2

) while other areas of tumour were negative. There was both cytoplasmic and nuclear staining for L-FABP.

There was a statistically significant association of less L-FABP immunoreactivity in poorly differentiated tumours (*χ*^2^=18.2, *P*<0.001). There was no relationship between L-FABP immunoreactivity and either tumour stage or overall survival.

### Colorectal adenomas

All the adenomas studied showed some L-FABP immunoreactivity with both nuclear and cytoplasmic immunoreactivity for L-FABP (Figure 2). There was a significant trend towards larger adenomas showing a smaller proportion of positively stained adenomatous epithelium (*χ*^2^=24.3, *P*<0.001, Table 7

**Table 7:** Proportion of L-FABP immunoreactive positive cells in colorectal adenomas

**L-FABP immunoreactivity**	**Small adenomas (*n*=6)**	**Medium adenomas (*n*=16)**	**Large adenomas (*n*=16)**
Negative	0	0	0
1–5%	0	0	12.5% (*n*=2)
6–25%	0	0	18.75% (*n*=3)
26–50%	0	100% (*n*=12)	62.5% (*n*=10)
51–75%	100% (*n*=6)	25% (*n*=4)	6.25% (*n*=1)
76–100%	0	0	0

).

### Normal colon

There was strong immunostaining of the surface epithelial cells of normal colon. Immunoreactivity for L-FABP extended down the upper half of the crypt epithelial cells while there was no staining of the basal crypt epithelial cells. There was consistent staining of both nuclei and cytoplasm. Other cell type including stromal cells, endothelial cells and chronic inflammatory cells did not stain in normal colon (Figure 2).

## DISCUSSION

A pathway of genetic changes occurring in colorectal cancer development has been well described (Ilyas *et al*, 1999). However, it is now clear that there are multiple genetic pathways of colorectal cancer development (Smith *et al*, 2002; Zhang *et al*, 2003) and that the ‘classic’ genetic model of colorectal development is probably only involved in a minority of colorectal cancers. The advent of proteomics now allows the changes in protein expression in colorectal cancer development and progression to be studied. In a recent overview of proteomics in cancer research we highlighted the key role of this technology in identifying changes in tumour protein expression (Lawrie *et al*, 2001b). We indicated both the rationale and the necessity to combine proteomics with other investigative techniques to allow the biological, pathological and clinical significance of those protein changes to be established (Lawrie *et al*, 2001b). This often requires the use of larger samples sizes and also allows the use of a wider range of clinical samples than may be used in proteomics. In this proof-of-principle study we have identified by proteomics the decrease of L-FABP in locally advanced colorectal cancer. We have then determined the pathological significance of altered L-FABP in a large series of colorectal cancers and delineated the changes that occur in L-FABP expression during colorectal cancer development and progression. The analysis of other alterations in protein expression in colorectal cancer that we identified is the subject of other manuscripts.

L-FABP has a role in several important physiological functions including intracellular signalling, control of cell division and cell differentiation and these functions are dysregulated in tumour development and progression. In normal hepatocytes, L-FABP mediates the stimulation of DNA synthesis and cell growth by long-chain fatty acids. (Glatz and van der Vusse, 1996). Evidence for the involvement of fatty acid binding proteins in cell growth and differentiation has been established through a series of studies on the action of chemical carcinogens on rat liver. It was found that the cellular content of L-FABP was markedly elevated during normal as well as carcinogen-induced mitotic activity of hepatocytes (Custer and Sorof, 1984, 1985) and that L-FABP was also specifically required in the induction of cell proliferation by two specific classes of hepatocarcinogenic peroxisome proliferators (Keler and Sorof, 1993; Khan and Sorof, 1994). A direct correlation was found between the relative affinities of the various peroxisome proliferators for L-FABP *in vitro* and their abilities to enhance mitogenesis (Sorof, 1994). Compared with other fatty acid binding proteins L-FABP has distinct fatty acid binding profile. In addition to acting as a fatty acid receptor L-FABP is the target protein for several genotoxic carcinogens (Sorof, 1994). Binding of these carcinogens to L-FABP promotes mitogenesis and this protein has also therefore been implicated in early tumour development (Khan and Sorof, 1994; Sorof, 1994).

L-FABP expression has previously been examined in the small intestine and colon of rats treated with 1,2-dimethylhydrazine, which produces a predictable pattern of both small and large bowel cancers (Davidson *et al*, 1993). This study found that there was a 50-fold decrease in L-FABP mRNA abundance in colon cancers compared with uninvolved mucosa. Immunocytochemical analysis also revealed decreased L-FABP staining in the colonic tumours, although mosaic clusters of L-FABP reactive tumour cells that showed both nuclear and cytoplasmic staining were often detected. The mosaic pattern of immunohistochemical staining for L-FABP was also observed in rat foetal small intestine and colon (Davidson *et al*, 1993). This led to the proposal that the expression of L-FABP was developmentally regulated and that L-FABP was involved in the promotion of normal intestinal differentiation. It was also suggested that the pathways involved in L-FABP regulation are disrupted in transformed and dedifferentiated enterocytes (Davidson *et al*, 1993). Our immunohistochemical results also demonstrate a patchy or ‘mosaic’ pattern of L-FABP positive tumour cells staining and are consistent with previous findings in chemically induced rat intestinal tumours (Davidson *et al*, 1993).

While 2D gel electrophoresis studies showed that L-FABP was consistently present in samples of normal colon mucosa, immunohistochemical studies highlighted its distribution and showed that L-FABP was strongly present in the surface epithelium and the crypt epithelium of the upper half of the crypt while it was not detected in the epithelial cells of the lower half of the crypt. This finding provides further support to the concept that L-FABP is a marker of colonocyte differentiation as previously observed in rat colon (Davidson *et al*, 1993).

The loss of L-FABP in colorectal neoplasms contrasts with the finding in other tumour types. In prostate cancer L-FABP mRNA and protein has been found to show markedly increased expression in both primary tumours and in prostate cancer derived cell lines (Das *et al*, 2001). Inhibition of L-FABP by antisense oligonucleotides in prostate cancer decreases tumour cell proliferation and promotes apoptosis. Expression of L-FABP has also previously been observed in primary liver cancer with L-FABP specifically localised to tumour cells. Higher frequency of L-FABP expression occurs in hepatoblastomas compared with hepatomas (Suzuki *et al*, 1990). Those observations in diverse types of tumours highlight tumour-specific expression patterns and presumably reflect tissue-specific regulatory mechanisms for L-FABP.

In conclusion, we have identified by proteomics the loss of L-FABP in colorectal cancer. We have determined the changes in L-FABP that occur during colorectal cancer development and progression, and have shown that its loss is an early stage event in colorectal tumour development. L-FABP was also found to be a marker of tumour differentiation in colorectal cancer. This study provides the basis for the evaluation of other differentially expressed proteins identified by colorectal cancer proteomics and evaluate them in the appropriate clinico-pathological context.
